# Left ventricular hypertrophy in hypertension: Is the electrocardiogram enough for risk stratification?

**DOI:** 10.1111/jch.14614

**Published:** 2022-12-20

**Authors:** Cesare Cuspidi, Elisa Gherbesi, Marijana Tadic

**Affiliations:** ^1^ Department of Medicine and Surgery University of Milano‐Bicocca Milan Italy; ^2^ Department of Cardio‐Thoracic‐Vascular Area Foundation IRCCS Cà Granda Ospedale Maggiore Policlinico Italy; ^3^ Department of Cardiology University Hospital “Dr. Dragisa Misovic – Dedinje”, Heroja Milana Tepica 1 Belgrade Serbia

**Keywords:** echocardiography, electrocardiogram, left ventricular hypertrophy

Left ventricular hypertrophy (LVH), diagnosed by electrocardiogram (ECG) or echocardiogram, (ECHO) is a powerful, independent predictor of cardiovascular (CV) events in different clinical settings.^1^ The sensitivity and specificity of ECG criteria, however, are recognized to be sub‐optimal, as shown by several studies having as reference LV mass evaluated at autopsy or by imaging techniques such as ECHO, computerized tomography, or cardiac magnetic resonance (CMR).^2^ Although the assessment of LV mass (LVM) by transthoracic ECHO represents the reference imaging tool for its established diagnostic accuracy in identifying LVH in hypertensive patients and improving CV risk stratification, the availability and the costs of this technique do not allow its routinary use in clinical practice.

Therefore, we read with great interest and attention the study by Park et al.^3^ aimed to investigate the value of LVH, phenotyped by ECG and ECHO criteria, in predicting the composite of major CV events including heart failure, myocardial infarction, peripheral artery disease, ischemic stroke, stroke, end stage renal disease (MACEs) or death among 1743 participants to Korean Hypertension Cohort study over a median 10‐year follow‐up period.

The key‐features of this long‐term prospective survey were the following: i) hypertensive patients with ECG‐defined LVH and normal LVM exhibited a significantly greater risk of MACEs and death than their counterparts without ECG‐ and ECHO‐defined LVH; ii) the detection of ECHO‐LVH improved CV risk estimation only in patients without ECG‐LVH, thus leading to the conclusion that the search for ECHO‐LVH would be useful only in patients without evidence of ECG‐LVH.

The prognostic value of ECG‐LVH, independent of imaging techniques, such as ECHO and CMR, has previously been demonstrated by some studies^4^, ^5^, ^6^ but not by others.^7^, ^8^

In a multi‐ethnic cohort of participants in the Multi‐Ethnic Study of Atherosclerosis (MESA) study without clinically detected CV disease, ECG‐LVH was associated with incident atrial fibrillation, regardless of CMR‐LVH.^5^ Findings provided by the Cardiovascular Health Study (CHS) showed that ECG‐LVH (HR = 1.68, 95%CI = 1.23, 2.28) and ECHO‐LVH (HR = 1.58, 95%CI = 1.17, 2.14) entailed an increased risk of stroke independently of each other, after adjusting for potential confounders.^6^ In the Pressioni Arteriose Monitorate E Loro Associazioni (PAMELA), an Italian population‐based study, ECG‐LVH without a concomitant increase in LVM index failed to predict CV death.^7^ Notably, the association between ECG‐ and ECHO‐LVH markedly increased the risk of CV fatal events independently of other risk factors known to predict mortality. Finally, a retrospective, population‐based cohort study of older individuals without overt CV disease, documented that ECHO, but not ECG markers of LVH predicted the long‐term risk of composite coronary events, heart failure, stroke, or death from any cause.^8^

In this complex scenario, the translational implications of Park's study^3^ supporting the indication to perform the echocardiogram only in patients with normal ECG, in consideration of its marginal additional prognostic value in patients with ECG‐LVH, need some considerations. First, the diagnosis of ECG‐LVH was based on three different criteria, but it is not clear which of these criteria played the most important diagnostic role for prognostic stratification, thus leaving this clinical aspect undefined. Second, the adoption of ECHO thresholds recommended by international guidelines (derived primarily from cohorts of Caucasian ethnicity) may have led to significant misclassification of LVH in a such sample of Asian hypertensive patients.^9^ Third, the diagnosis of ECHO‐LVH was exclusively made by indexing LVM by body surface area without performing a sensitivity analysis based on the indexation for height^2.7^ more appropriate in identifying LVH in overweight and obese individuals in which ECG criteria are less performing.^10^ Fourth, factors associated with diagnostic discrepancy between ECG‐ and ECHO‐LVH criteria have not been addressed, as well as gender‐related differences in the association between LVH and outcome.^11^

Last but not least, it is worth noting that beyond mere identification of LVH, ECHO provides a variety of useful information (i.e., LV function, valve disease, left atrial, and aortic root dimensions) for the clinical management both in hypertensive patients with and without ECG‐LVH.

## AUTHOR CONTRIBUTIONS

Cesare Cuspidi – writing the article. Elisa Gherbesi – detailed review with constructive remarks that substantially changed the article. Marijana Tadic – detailed review with constructive remarks that substantially changed the article.
